# Evidence on Integrating Pharmacokinetics to Find Truly Therapeutic Agent for Alzheimer's Disease: Comparative Pharmacokinetics and Disposition Kinetics Profiles of Stereoisomers Isorhynchophylline and Rhynchophylline in Rats

**DOI:** 10.1155/2019/4016323

**Published:** 2019-02-03

**Authors:** Chunyuan Zhang, Xu Wu, Yanfang Xian, Lin Zhu, Ge Lin, Zhi-Xiu Lin

**Affiliations:** ^1^School of Chinese Medicine, Faculty of Medicine, The Chinese University of Hong Kong, Hong Kong SAR, Hong Kong; ^2^Laboratory of Molecular Pharmacology, Department of Pharmacology, School of Pharmacy, Southwest Medical University, Luzhou 646000, Sichuan, China; ^3^School of Biomedical Sciences, Faculty of Medicine, The Chinese University of Hong Kong, Hong Kong SAR, Hong Kong

## Abstract

Isorhynchophylline (IRN) and rhynchophylline (RN), a pair of stereoisomers, are tetracyclic oxindole alkaloids isolated from* Uncaria rhynchophylla*, a commonly used Chinese medicinal herb. These two compounds have drawn extensive attention due to their potent neuroprotective effects with promising therapeutic potential for the treatment of Alzheimer's disease (AD). However, IRN and RN can interconvert into each other* in vivo* after oral administration. The present study aimed to elucidate the pharmacokinetic profiles and disposition kinetics of the administered and generated stereoisomers in the brain and cerebrospinal fluid (CSF) after oral administration of equal dose of IRN or RN to rats. Our study demonstrated that after oral administration, RN showed significantly higher systemic exposure (6.5 folds of IRN,* p* < 0.001) and disposition in the brain (2.5 folds of IRN,* p* < 0.01) and CSF (3 folds of IRN,* p* < 0.001) than IRN. The results indicated that interconversion between IRN and RN occurred. Notably, regardless of the orally administered IRN or RN, RN would always be one of the major or predominant forms present in the body. Our results provided sound evidence supporting further development of RN as a potential therapeutic agent for the treatment of AD. Moreover, the present study sets a solid example that integrating pharmacokinetics is crucial to identify the truly therapeutic agent.

## 1. Introduction

Alzheimer's disease (AD) is the most common form of neurodegenerative disease in the elderly population [1, 2]. Alkaloids-containing herbal extracts have been widely used as therapeutic agents in traditional medicine for thousands of years [3]. The use of naturally occurring alkaloids as therapeutic agents for AD treatment has drawn extensive attention, and the U.S. Food and Drug Administration has recently approved two alkaloids, i.e., galantamine and rivastigmine, which act as cholinesterase inhibitors, for the treatment of AD [4, 5].

**Figure 1:**
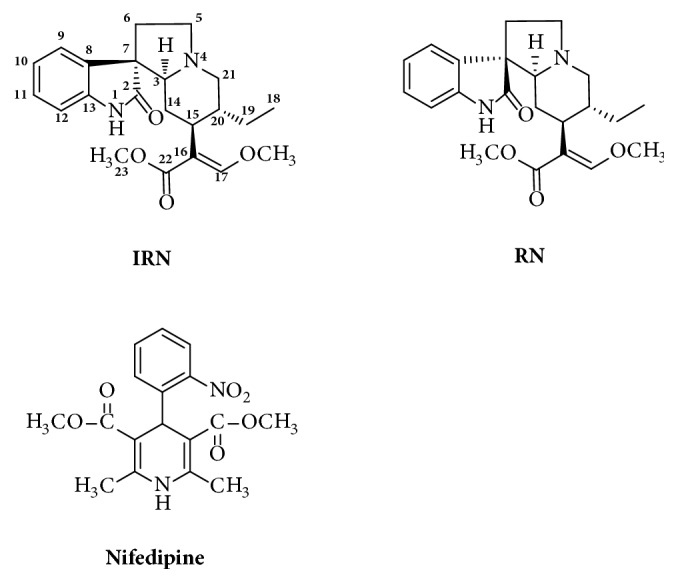
Chemical structures of isorhynchophylline (IRN), rhynchophylline (RN) and nifedipine (internal standard, IS).

*Uncaria rhynchophylla* (Gou-Teng in Chinese) has been demonstrated as a promising herbal medicine for the treatment of AD. The extract of* U. rhynchophylla* has been reported to have potent antiaggregation effects on amyloid-*β* proteins [6] and was demonstrated to improve cognitive deficits induced by D-galactose in mice [7]. The major active components in* U. rhynchophylla* have been revealed to be oxindole alkaloids. Isorhynchophylline (IRN) and rhynchophylline (RN) (Figure 1) are tetracyclic oxindole alkaloids accounting for more than 43% of the total alkaloid content in* U. rhynchophylla* [8] and have been regarded as the major pharmacologically active components in the herb [9–11]. Investigations of the pharmacological effects of IRN and RN have revealed that they could exert beneficial effects on AD. Recent studies conducted by our group have indicated that IRN could rescue PC12 cells from amyloid-*β*-induced apoptosis [12] and also exhibited neuroprotective effect in amyloid-*β*-treated PC12 cells [13]. Both IRN and RN were able to exert neuroprotective effect by protecting amyloid-*β*-treated PC12 cells from cell death [14]. Furthermore, IRN could ameliorate cognitive deficits, enhance the antioxidative status, and reduce inflammation via inhibition of the NF-*κ*B signaling pathway in the brain tissues of the D-galactose-induced mice [15]. IRN was also able to improve cognitive deficits via the inhibition of neuronal apoptosis and tau protein hyperphosphorylation in the hippocampus of the amyloid-*β*-treated rats [16]. More recently, other research groups identified RN as an inhibitor of tyrosine kinase EphA4 receptor and demonstrated that RN could restore the synaptic impairment in the transgenic mouse models of AD [17] and could ameliorate amyloid-*β*-induced perturbation of hippocampal CA1 neuronal activity [18].

Because both IRN and RN are promising candidates for further development into therapeutic agents for AD, understanding their disposition kinetics in the brain and cerebrospinal fluid (CSF) and plasma levels of IRN and RN is important. In addition, IRN and RN are a pair of stereoisomers at C7 chiral position. Interconversion between IRN and RN was firstly discovered by Wenkert et al. in 1959 [19], and this phenomenon has been observed both* in vitro* and* in vivo *[20–29]. It is worth noting that stereoconfiguration at C7 position of IRN and RN may lead to differences in their pharmacokinetics. Therefore, knowledge of the difference in pharmacokinetic profiles and disposition kinetics of IRN and RN and the epimerization between them is critical for further development of their therapeutic usage. However, previous pharmacokinetic studies on IRN and RN were only conducted separately [20–22]. The most recent stereoselective pharmacokinetic study on IRN and RN failed to reveal the disposition kinetics in the brain and the pharmacokinetic profiles of generated stereoisomers [27, 29]. In the present study, we aimed to elucidate the kinetic profiles of the orally administered and metabolically generated stereoisomers in the brain, CSF, and plasma of rats via studying in parallel both IRN and RN.

## 2. Materials and Methods

### 2.1. Chemicals and Reagents

IRN (purity ≥ 98%) and RN (purity ≥ 98%) were purchased from Chengdu Mansite Pharmaceutical Co. Ltd. (Chengdu, Sichuan, China). Nifedipine (purity ≥ 98%), used as internal standard (IS, Figure 1), was purchased from Sigma-Aldrich Chemical Co. (St. Louis, MO, US). Heparin and Tween 80 were purchased from Sigma-Aldrich Chemical Co. (St. Louis, MO, US). Acetonitrile of HPLC grade was obtained from Duksan Pure Chemicals (South Korea). All other compounds and reagents not listed were of analytical grade.

### 2.2. Investigation of the Kinetic Profiles of Administered and Generated Stereoisomers in the Brain, CSF, and Plasma of the Rats

Male Sprague-Dawley (SD) rats were provided by the Laboratory Animal Service Center of The Chinese University of Hong Kong, Hong Kong, China. Animals were housed in cages during the study period under standard conditions of temperature, humidity, and light. All animal experiments were approved by the Animal Ethics Committee of The Chinese University of Hong Kong.

One day before the experiment, the male SD rats (160-200 g) were fasted overnight with free access to water. On the following day, the rats were randomly divided into two groups (n = 18 per group) for oral administration of either IRN or RN at a dosage of 20 mg/kg. The dosage was the same as used in the previous pharmacodynamics studies [15, 16]. The suspension solutions of IRN and RN were prepared in 5% Tween 80, respectively. The SD rats in the two groups were orally administered at a dose of 20 mg/kg IRN or RN and sacrificed at 15, 30, 60, 90, 120, and 180 min postdosing (n = 3 per time interval). The rats were exsanguinated by cardiac puncture under anesthesia, followed by collecting their CSF (about 100 *μ*L) through a single puncture in the cisterna magna. Plasma was obtained by centrifugation of blood at 6,000 × g for 8 min at 4°C. After blood collection, the animals were perfused transcardially with normal saline until total blood volume was removed. Then, the whole brain was promptly collected, and the surface water was blotted dry with Kimwipes (Kimberly-Clark™). Blood vessels and meninges were carefully removed with forceps and the brain was weighted. All samples were stored at −20°C until analysis.

### 2.3. Brain, CSF, and Plasma Sample Preparations

Brain homogenate was freshly prepared by homogenizing brain tissues in normal saline (1:2, w/v). Homogenization was conducted in an ice bath using the IKA T10 basic ULTRA-TURRAX Homogenizer for 20–30 s.

The biological samples including brain homogenate, CSF, and plasma (50 *μ*L each) were deproteinized by adding 150 *μ*L of acetonitrile (containing 20 ng/mL IS). After vortex mixing for 1 min, the mixture was centrifuged (20,000 × g) for 10 min at 4°C. After centrifugation, an aliquot (5 *μ*L) of supernatant was subjected to the LC-MS/MS analysis.

### 2.4. Quantitation of IRN and RN in Various Biological Samples by LC-MS/MS

Quantitation of IRN and RN in biological samples was performed on an Agilent 1290 Infinity LC system coupled with an Agilent 6460 Triple Quad tandem mass spectrometry with an ESI interface system (Agilent Technologies Inc., US). The chromatographic separation was achieved on ZORBAX Eclipse Plus C18 column (2.1 × 100 mm, 1.8 *μ*m, Agilent) maintained at 40°C temperature at a flow rate of 0.3 mL/min. A gradient mobile phase consisting of 0.1% formic acid in acetonitrile (A) and 0.1% formic acid in water (B) was eluted as follows: 0–1 min, A, 15–20%; 1–5 min, A, 20–25%; 5–7 min, A, 25–95%; 7–10 min, A held at 95% and returned to the initial condition (acetonitrile-water ratio 15 : 85) for a 3 min equilibration. The temperature of the auto-sampler was kept at 4°C.

The MS/MS system was operated in positive mode and multiple reaction monitoring (MRM) mode under the following operation parameters: gas temperature, 300°C; gas flow, 5 L/min; nebulizer gas, 45 psi; capillary voltage, 3500 V; Fragmentor, 150 V (for IRN and RN), 76 V (for IS); cell accelerator, 4 V; Dwell, 80 ms. IRN and RN were monitored at the* m/z* 385 ([M+H]^+^) to* m/z* 160 ([M+H-225]^+^) transition and the IS at the* m/z* 347 ([M+H]^+^) to* m/z* 315 ([M+H-32]^+^) transition. All the data were processed using Agilent MassHunter Workstation Software Quantitative Analysis Version B.07.00/Build 7.0.457.0 (Agilent).

### 2.5. Data Analysis

Kinetic parameters were calculated using the WinNonlin software (Version 4.0, Pharsight Corp, Mountain View, CA, US) employing a noncompartmental model approach. Epimerization ratio was calculated by AUC_(generated  stereoisomer)_/(AUC_(generated  stereoisomer)_ + AUC_(administered  stereoisomer)_) × 100%. The brain-to-plasma partition coefficient (K_p_, Brain) was calculated by AUC_Brain_/AUC_Plasma_, and the CSF-to-plasma partition coefficient (K_p_, CSF) was calculated by AUC_CSF_/AUC_Plasma_.

All the data in the study were expressed as mean ± SEM. Statistical analyses were performed using GraphPad Prism 6.0 (GraphPad Software Inc, San Diego, CA, US) using two tailed unpaired* t*-test for two groups comparison. Statistical significance was set at *p* value less than 0.05.

## 3. Results

### 3.1. Disposition Kinetics of Administered Stereoisomers in the Rat Brain, CSF, and Plasma

**Figure 2:**
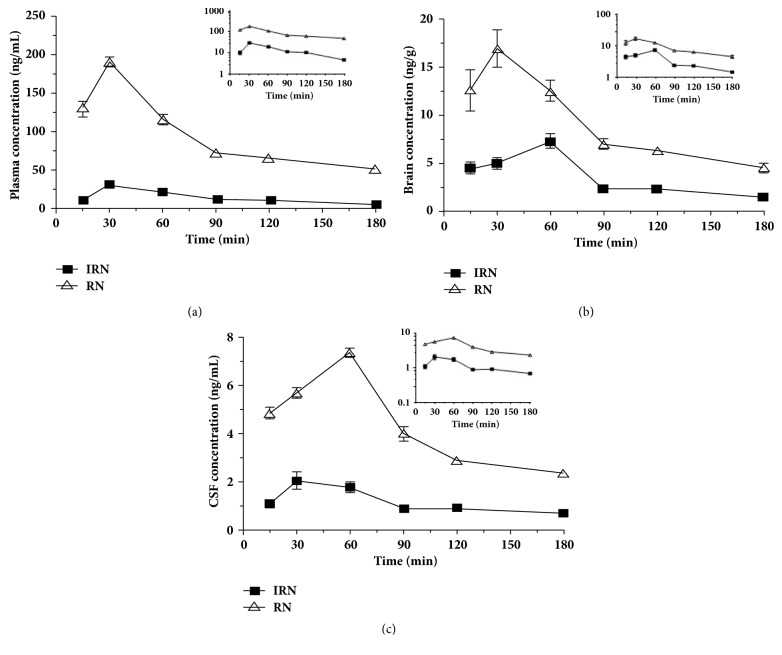
Mean concentration-time profiles of the administered IRN and RN in the rat plasma (a), brain (b) and CSF (c) after oral administration (The semi-logarithmic plots were inserted in the corresponding figures).

**Figure 3:**
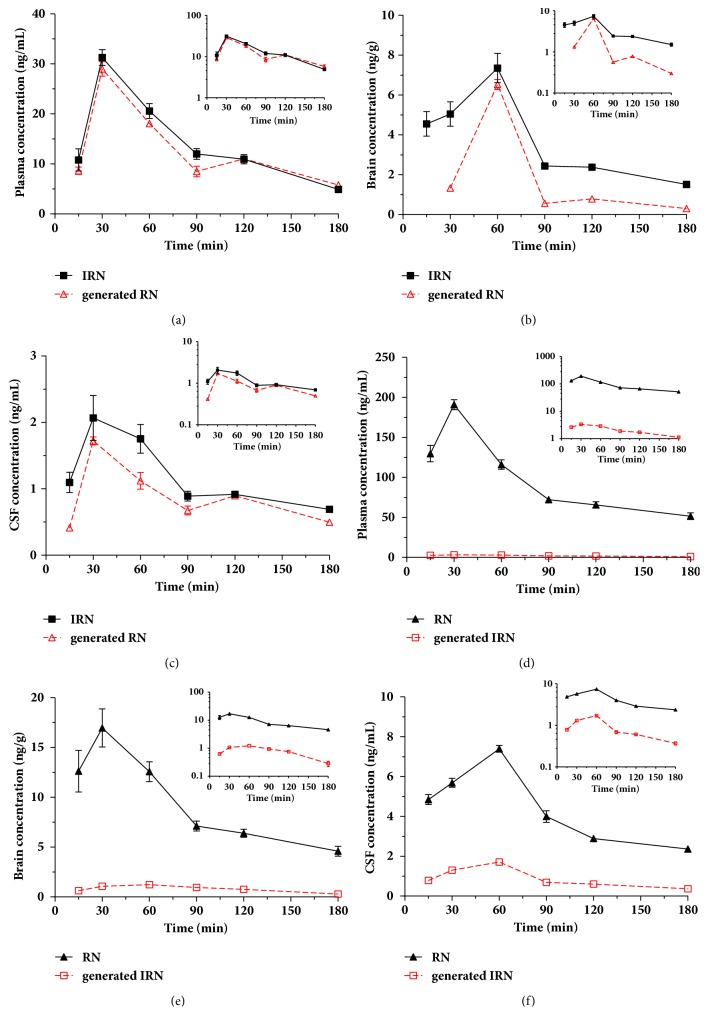
Mean concentration-time profiles of the administered IRN and generated RN in the rat plasma (a), brain (b) and CSF (c) after oral administration of IRN at 20 mg/kg. Concentration-time profiles of the administered RN and generated IRN in the rat plasma (d), brain (e) and CSF (f) after oral administration of RN at 20 mg/kg. (Semi-logarithmic plots were inserted in the corresponding figures).

The developed LC-MS/MS method that could separate the stereoisomers IRN and RN was successfully applied for the quantitation of both stereoisomers in all collected biological specimens. The typical MRM chromatograms of the blank biological specimen, blank biological specimen spiked with both analytes and samples obtained after oral administration of IRN or RN were shown in the supplementary data. The MRM chromatograms of blank plasma, brain tissues, and CSF (Figure S1) did not show any interfering peaks or signal at the retention times of the target analytes IRN (4.59 min) and RN (5.44 min). The calibration curves were generated by plotting the peak area ratio of IRN or RN to IS against the concentration of IRN or RN. The regression equations, linearity ranges, and correlation coefficients for individual standard curves are summarized in Table 1. The mean concentration-time profiles of administered and generated stereoisomers in the rat plasma, brain, and CSF after oral administration of IRN or RN at 20 mg/kg were obtained (Figures 2 and 3), and kinetic parameters were determined (Table 2).

The results showed that, after oral administration of equal dosage of IRN or RN, both rapidly reached their peak concentration in plasma at 30 min (Figure 2(a)), while the absorption and systemic exposure of the administered RN (C_max_: 190.87 ± 6.34 ng/mL; AUC_Plasma_: 16382.06 ± 269.22 ng·min/mL) were significantly higher than those of the administered IRN (C_max_: 31.29 ± 1.59 ng/mL; AUC_Plasma_: 2483.43 ± 83.83 ng·min/mL). On the other hand, the elimination of IRN was significantly faster than that of RN (t_1/2_: 64.31 ± 3.19 min* vs.* 129.53 ± 9.30,* p *< 0.01) (Figure 2(a) and Table 2). Similarly, the overall brain exposure of RN (AUC_Brain_: 1587.03 ± 127.82 ng·min/g and C_max_: 16.96 ± 1.92 ng/mL) was also significantly higher than IRN (AUC_Brain_: 627.37 ± 43.31 ng·min/g and C_max_: 7.35 ± 0.74 ng/mL) (Figure 2(b) and Table 2). Moreover, RN also had significantly greater exposure in CSF, with AUC_CSF_ and C_max_ being more than 3-fold of those of IRN (*p* < 0.001) (Figure 2(c) and Table 2).

All the findings revealed that after oral administration of IRN or RN at the same dose, the systemic exposure of RN was significantly higher (about 6.5-fold,* p *< 0.001) than that of IRN due to its significantly greater oral absorption and slower clearance. Consequently, the higher plasma concentrations of RN also resulted in significantly higher exposure in both brain and CSF when comparing with that of IRN (*p* < 0.01).

### 3.2. Epimerization and Disposition Kinetics of Generated Stereoisomers in the Rat Brain, CSF, and Plasma

After oral administration of the same dose, at least 47% of the administered IRN were metabolically converted to RN and then distributed into plasma (47.54 ± 0.22%), brain (32.21 ± 1.22%), and CSF (43.09 ± 0.92%) (Figures 3(a), 3(b), and 3(c) and Table 2). The systemic exposure of the administered IRN and generated RN in plasma was similar (epimerization ratio: 47.54 ± 0.22%), while the exposure in the brain (AUC_Brain_) and CSF (AUC_CSF_) of the generated RN was about half of the administered IRN. Whereas, after oral administration of RN, significantly less conversion to IRN occurred and approximately 2.20%, 8.23%, and 16.91% of the generated IRN were found in the plasma, brain, and CSF, respectively (Figures 3(d), 3(e), and 3(f) and Table 2). The results demonstrated that after oral administration of the same dosage of IRN or RN, the plasma concentrations of RN were always higher, with its systemic exposure significantly higher than that of IRN after administration of RN.

Moreover, for both IRN and RN administration, the patterns of the mean concentration-time profiles in plasma, CSF, and brain of the administered and generated stereoisomers were all comparable (Figure 3). For instance, after oral administration of RN, similar T_max_ (30 min) and t_1/2_ (129.53 ± 9.0 (RN)* vs.* 116.60 ± 13.58 min (IRN)) values were observed for the administered RN and the generated IRN. These results indicated that the concentrations of the generated stereoisomers altered along with the changes of the concentrations of the administered stereoisomers in the plasma followed by the brain.

On the other hand, it was noted that after oral administration of RN, the K_p,Brain_ and K_p,CSF_ values of the generated IRN were significantly higher than those of administered RN (*p* < 0.001), and the epimerization ratios of RN in the brain (8.23 ± 0.56%) and CSF (16.91 ± 0.51%) were significantly higher than that in the plasma (2.2 ± 0.04%), indicating that IRN had a better capability than RN to penetrate into the brain. In addition, for the oral administration of IRN, although similar systemic exposure of the administered IRN and the generated RN was determined, the exposures in the brain (AUC_Brain_) and CSF (AUC_CSF_) of the administered IRN were significantly higher than that of the generated RN. These data further confirmed the higher permeability of IRN than RN into the brain. These results further indicated that (1) after oral administration of equal dosage of IRN or RN, both were absorbed rapidly into the systemic circulation and subsequently reached the brain via penetration through the blood-brain barrier (BBB); (2) the systemic exposure of RN was always pronounced with either similar to (after IRN administration) or significantly higher than that of IRN (after RN administration) due to significantly more extensive conversion of IRN to RN than that of RN to IRN, and significantly greater oral absorption and slower clearance of RN; and (3) after IRN administration, the concentrations of IRN in brain were significantly higher than that of RN due to the significantly higher permeability of IRN into the brain.

## 4. Discussion

Although both IRN and RN exhibited potent neuroprotective effects with promising therapeutic potential for the treatment of AD, differences in their pharmacological action exist. RN but not IRN was identified as a potent inhibitor of EphA4 to restore the synaptic impairment in AD [17]. On the other hand, IRN showed significant neuroprotective activity against glutamate-induced HT22 cell injury, while RN only displayed weak effect [25]. Meanwhile, it should be noted that IRN and RN can be interconverted* in vivo*. This indicates that the stereoselective pharmacokinetic study of these stereoisomers is crucial for identifying truly therapeutic agent for AD. Therefore, in the present study, we for the first time simultaneously investigated the kinetic profiles of the administered and generated stereoisomers in the brain, CSF, and plasma of the rats after oral administration of equal dose of IRN or RN.

We firstly observed significant stereoselective pharmacokinetics and epimerization of IRN and RN in the rats. After oral administration, both IRN and RN absorbed rapidly and reached their peak concentration in the plasma within 30 min. However, the systemic exposure (AUC_Plasma_) of IRN was 6.5-fold lower than that of RN. These results were in good agreement with the data reported previously for the intact IRN and RN orally administered [29], where it was found that the bioavailability of RN (25.9 ± 8.7%) was 7.8-fold higher than that of IRN (3.3 ± 0.8%). Recent studies showed that this was mainly attributable to the stereoselective metabolism in liver. IRN was much more favorable to be metabolized than RN in the rat liver microsomes, and this stereo selectivity in hepatic metabolism of two stereoisomers was mainly mediated by CYP3A4 [28]. Moreover, the favorable conversion of IRN to RN also accounted for the low bioavailability of IRN [27]. The epimerization ratio of IRN to RN was 47.54 ± 0.22% in plasma while the epimerization ratio of RN to IRN is only 2.20 ± 0.04%. However, the AUC_Plasma_ of generated RN after IRN administration was much lower (2249.95 ± 63.83* vs.* 16382.06 ± 269.22 ng·min/mL) than that of RN after RN administration, suggesting that the stereoselective interconversion made a smaller contribution to the difference in bioavailability of IRN and RN. Overall, RN might be a better choice for further development into an anti-AD agent due to its high bioavailability.

Importantly, in this study, the kinetic profiles of the metabolic converted stereoisomers (the generated RN from IRN and the generated IRN from RN) in the rat brain, CSF, and plasma were simultaneously elucidated after oral administration of IRN or RN. It is interesting to find that, regardless of the orally administered IRN or RN, RN was always one of the major or predominant forms present in the body. Our results showed that after administration of IRN, approximately half of IRN was metabolically converted to RN as identified in the plasma, brain, and CSF. On the other hand, after administration of RN, about 2.20%, 8.23%, and 16.91% of the generated IRN were found in the plasma, brain, and CSF, respectively; and both AUC_Brain_ and AUC_CSF_ of the generated IRN were significantly lower than those of RN. It has been reported that the interconversion between IRN and RN was partially based on their pK_a_ values. RN (pK_a_ 6.32) is a stronger base than IRN (pK_a_ 5.20) [30]. In the acidic condition, RN predominates because it could be stabilized by hydrogen bonding between the protonated nitrogen and oxindole carbonyl [30]. In addition, according to a previous report, after intravenous administration of equal dosage of IRN or RN, there was no significant difference between IRN and RN in the systemic exposure [29]. Therefore, the conversion between IRN and RN might take place in the GI tract, especially in the acidic compartment (pH 3.0-3.8) like stomach [31]. Moreover, epimerases, which are ubiquitous and catalyze versatile epimerization in all organisms [32], could also interconvert IRN into RN by catalyzing an epimerization on the C7 stereocenter. The significant difference in epimerization ratios between IRN and RN might also be due to the stereospecificity of epimerization.

Our results also revealed that IRN had significantly higher K_p,Brain_ and K_p,CSF_ than that of RN (*p *< 0.01), suggesting that IRN had better brain penetration capability. This was consistent with a previously reported study of* in vitro *BBB permeability, in which the BBB permeability of IRN and RN was measured using a coculture model composed of three types of cells, namely endothelial cells, pericytes, and astrocytes. Both IRN and RN could pass through brain endothelial cells, while IRN showed a 1-fold higher BBB permeability than RN [33]. Additionally, the membrane permeability of RN was affected by P-gp efflux transporter [27, 34]. Overall, our study for the first time confirmed the* in vivo* BBB permeability of IRN and RN, with IRN more permeable than RN. However, despite the higher BBB permeability of IRN, its BBB penetration was still lower than RN due to the poor bioavailability.

The therapeutic effects of both IRN and RN in AD have been recently investigated in different animal models [15, 17, 18]. Fu et al. found that oral administration of RN (50 mg/kg/day for 3-4 weeks) restored the impaired long-term potentiation in the hippocampus of APP/PS1 transgenic mice [17], while Xian et al. showed that oral administration of IRN (20 or 40 mg/kg for 3 weeks) ameliorated cognitive deficits induced by A*β*_25-35_ in rats [15]. It should be noted that although both IRN and RN were capable of exerting anti-AD effect* in vivo*, the present kinetic study provided new evidence supporting that RN was more suitable than IRN for further development into an anti-AD agent, mainly due to the following: (1) low bioavailability of IRN; (2) high epimerization ratio of IRN; and (3) after oral administration of an equal dose of RN or IRN, the overall exposure of RN in the plasma, brain, and CSF was much higher than that of IRN.

## 5. Conclusion

The present study for the first time simultaneously probed the plasma pharmacokinetics and disposition kinetics of administered and generated stereoisomers in the brain and CSF after oral treatment of equal dose of IRN or RN in rats. Our findings unambiguously demonstrated that after oral administration, RN exhibited markedly higher systemic exposure and disposition in the brain and CSF than IRN. Moreover, with the same oral dose, regardless of the orally administration of IRN or RN, RN would always be one of the major or predominant forms present in the body. The results obtained from the present study provided sound experimental evidence to support further development of RN into a potential therapeutic agent for the treatment of AD. Our present study also set a good example on integrating pharmacokinetics for identifying the truly therapeutic agent.

## Figures and Tables

**Table 1:** Regression equations, linearity ranges, and correlation coefficients of the calibration curves.

Analyte	Biological specimen	Regression equation	Linear ranges (ng/mL)	Correlation coefficient
IRN	Brain	*y* = 0.2100*x *+ 0.0016	0.1–5.0	0.9996
	CSF	*y* = 0.3187*x *+ 0.0042	0.1–5.0	0.9994
	Plasma	*y* = 0.7395*x *– 0.0137	1.0–50.0	0.9991

RN	Brain	*y* = 1.5308*x *+ 0.0081	0.5–10.0	0.9992
		*y* = 1.5308*x *+ 0.0081	0.1–5.0	0.9991
	CSF	*y* = 1.3104*x *– 0.0124	0.5–10.0	0.9994
		*y* = 1.3029*x *– 0.0002	0.1–5.0	0.9991
	Plasma	*y* = 1.4469*x *– 0.2262	25.0–500.0	0.9992
		*y* = 0.6529*x *+ 0.1385	1.0–50.0	0.9996

**Table 2:** Kinetic parameters of the administered and generated stereoisomers in the brain, CSF, and plasma (mean ± SEM, n = 3 per time interval).

Samples	Parameters	IRN (20 mg/kg, *p.o.*)		RN (20 mg/kg, *p.o.*)	
		IRN	Generated RN	RN	Generated IRN
Plasma	AUC_0-180 min_ (ng·min/mL)	2483.43 ± 83.83	2249.95 ± 63.83	16382.06 ± 269.22^*∗∗∗*^	369.30 ± 7.65^†††^
	C_max_ (ng/mL)	31.29 ± 1.59	29.00 ± 1.44	190.87 ± 6.34^*∗∗∗*^	3.35 ± 0.06^†††^
	T_max_ (min)	30.00	30.00	30.00	30.00
	*t*_1/2_ (min)	64.31 ± 3.19	89.65 ± 14.09	129.53 ± 9.30^*∗∗*^	116.60 ± 13.58
	Epimerization ratio (%)	—	47.54 ± 0.22	—	2.20 ± 0.04^###^

Brain	AUC_0-180 min_ (ng·min/g)	627.37 ± 43.31	296.63 ± 9.49^*∗∗*^	1587.03 ± 127.82^*∗∗*^	140.76 ± 1.50^†††^
	K_p,Brain_^b^	0.2530 ± 0.0187	0.1322 ± 0.0068^*∗∗*^	0.0967 ± 0.0063^*∗∗*^	0.3814 ± 0.0065^†††^
	C_max_ (ng/g)	7.35 ± 0.74	6.53 ± 0.25	16.96 ± 1.92^*∗∗*^	1.22 ± 0.01^††^
	Epimerization ratio (%)	—	32.21 ± 1.22	—	8.23 ± 0.56^###^

CSF	AUC_0-180 min⁡_ (ng·min/mL)	204.25 ± 16.53	153.93 ± 7.87	742.69 ± 12.80^*∗∗∗*^	151.15 ± 5.09^†††^
	K_p,CSF_^c^	0.0821 ± 0.0050	0.0683 ± 0.0016	0.0453 ± 0.0002^*∗∗*^	0.4092 ± 0.0098^†††^
	C_max_ (ng/mL)	2.14 ± 0.34	1.72 ± 0.06	7.39 ± 0.17^*∗∗∗*^	1.71 ± 0.05^†††^
	Epimerization ratio (%)	—	43.09 ± 0.92	—	16.91 ± 0.51^###^

Epimerization ratio was calculated by AUC_(generated  stereoisomer)_/(AUC_(generated  stereoisomer)_ + AUC_(administered  stereoisomer)_) × 100%. The brain-to-plasma partition coefficient (K_p,Brain_) was calculated by AUC_Brain_/AUC_Plasma_, and the CSF-to-plasma partition coefficient (K_p,CSF_) was calculated by AUC_CSF_/AUC_Plasma_.

^*∗∗*^ *p *< 0.01, ^*∗∗∗*^*p* < 0.001, compared with IRN group (administered with 20 mg/kg IRN).

^††^ *p *< 0.01, ^†††^*p *< 0.001, compared with RN group (administered with 20 mg/kg RN).

^###^ *p* < 0.001, compared with generated RN group (administered with 20 mg/kg IRN).

## Data Availability

The current data used to support the findings of this study are included within the article.

## Abbreviations

AD: Alzheimer's disease
BBB: Blood-brain barrier
CSF: Cerebrospinal fluid
IRN: Isorhynchophylline
IS: Internal standard
MRM: Multiple reaction monitoring
RN: Rhynchophylline
SD: Sprague-Dawley.

## Supplementary Materials

Supplementary Materials The typical MRM chromatograms of the blank biological specimen, blank biological specimen spiked with both analytes and samples obtained after oral administration of IRN or RN were shown in Figure S1.
