# Supplementary figures and images for: Evidence on Integrating Pharmacokinetics to Find Truly Therapeutic Agent for Alzheimer's Disease: Comparative Pharmacokinetics and Disposition Kinetics Profiles of Stereoisomers Isorhynchophylline and Rhynchophylline in Rats
Source: Evid Based Complement Alternat Med. 2019 Feb 3;2019:4016323. doi: 10.1155/2019/4016323 (PMC6377964; doi:10.1155/2019/4016323)

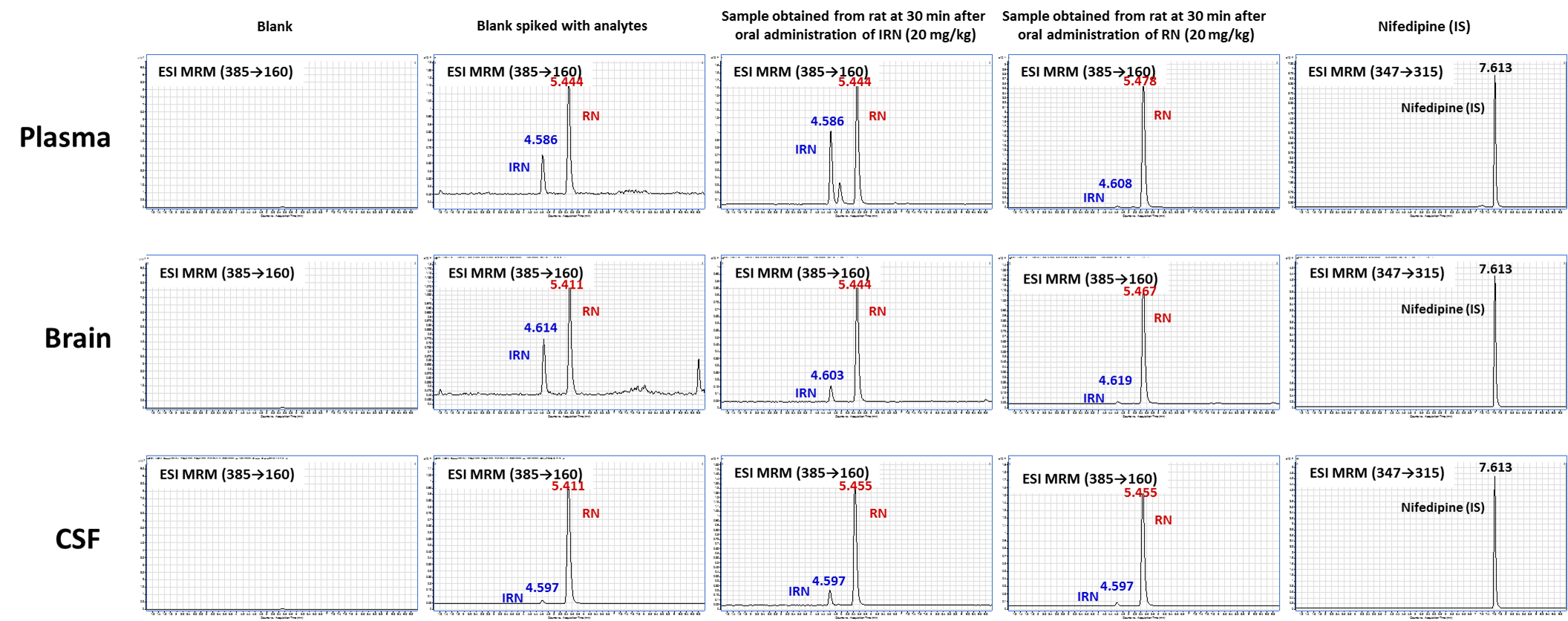

Supplement: Supplementary Materials — The typical MRM chromatograms of the blank biological specimen, blank biological specimen spiked with both analytes and samples obtained after oral administration of IRN or RN were shown in Figure S1. [file 4016323.f1.docx]
